# Preventive effects of coixol, an active compound of adlay seed, in NGF-differentiated PC12 cells against beta-amyloid_25-35_-induced neurotoxicity

**DOI:** 10.2478/abm-2024-0030

**Published:** 2024-10-31

**Authors:** Jan-Yow Chen, Chien-Yu Li, Mei-Chin Mong, Mei-Chin Yin

**Affiliations:** Department of Internal Medicine, China Medical University Hospital, Taichung 404328, Taiwan; Department of Neurosurgery, Asia University Hospital, Taichung 413305, Taiwan; Department of Food Nutrition and Health Biotechnology, Asia University, Taichung 413305, Taiwan; Department of Medical Research, China Medical University Hospital, China Medical University, Taichung 404328, Taiwan; Office of Research and Development, Asia University, Taichung 413305, Taiwan

**Keywords:** Abeta_25-35_, Ca^2+^ homeostasis, coixol, inflammatory stress, NGF-differentiated PC12 cells, oxidative stress

## Abstract

**Background:**

The health benefits of coixol, an active compound of adlay seed, have attracted certain attention. Adlay seed is often adopted in traditional Chinese medicine for the treatment of various inflammatory disorders. Thus, it is hypothesized that coixol could protect neuronal cells.

**Objectives:**

The preventive effects of coixol against Abeta_25-35_-induced damage in nerve growth factor-differentiated PC12 cells were explored.

**Methods:**

Differentiated PC12 cells were treated with coixol at 0.125 μM, 0.25 μM, 0.5 μM, 1 μM, and 2 μM for 48 h. Then, cells were further exposed to Abeta_25-35_ at 20 μM for 24 h.

**Results:**

Coixol treatments at 0.25–2 μM exhibited antiapoptotic effect via increasing Bcl-2 mRNA expression, mitochondrial membrane potential, and Na^+^-K^+^ ATPase activity as well as decreasing Bax mRNA expression, caspase-3 activity, and intracellular Ca^2+^ release. In addition, coixol treatments at 0.25–2 μM alleviated oxidative and inflammatory responses via lowering reactive oxygen species level, increasing glutathione content, promoting the activity of glutathione peroxidase, glutathione reductase, and catalase, decreasing the generation of tumor necrosis factor-α, interleukin (IL)-1β, IL-6, and prostaglandin E_2_. Furthermore, coixol treatments at 0.25–2 μM diminished intracellular Ca^2+^ release, and restricted nuclear factor kappa B-binding activity and phosphorylation of p65 and p38. Coixol treatments at 0.5–2 μM increased protein generation of nuclear factor E2-related factor 2, and limited protein production of inducible nitric oxide synthase and receptor of advanced glycation end product.

**Conclusion:**

Our novel findings suggested that coixol was a compelling agent against beta-amyloid peptide-induced neurotoxicity.

Coixol (6-methoxy-1,3-benzoxazol-2(3H)-one, **Figure 1A**) is an active polyphenolic compound presented in the *seeds of* adlay (*Coix lachryma-jobi* L.) [1]. Adlay seed (**Figure 1B**) is commonly used as a cereal food in several Asian countries such as Korea and China [2]. In addition, adlay seed is often adopted in traditional Chinese medicine for the treatment of rheumatism, neuralgia, and other inflammatory diseases [3]. So far, the health benefits of coixol have attracted certain attention. Wang et al. [4] reported that coixol possessed both antioxidant and antimicrobial activities. The study of Amen et al. [5] revealed that coixol displayed inhibitory actions against melanogenesis. Hameed et al. [6] found that coixol promoted glucose-stimulated insulin via mediating cAMP signaling pathway. Hu et al. [7] reported that coixol could suppress nuclear factor kappa B (NF-κB) and p38 mitogen-activated protein kinase (MAPK) pathways to ameliorate lipopolysaccharide-induced inflammatory reactions. Those previous studies strongly suggest that coixol is an agent with remarkable medicinal and nutritional values. Thus, it is highly possible that coixol could protect neuronal cells.

**Figure 1.**
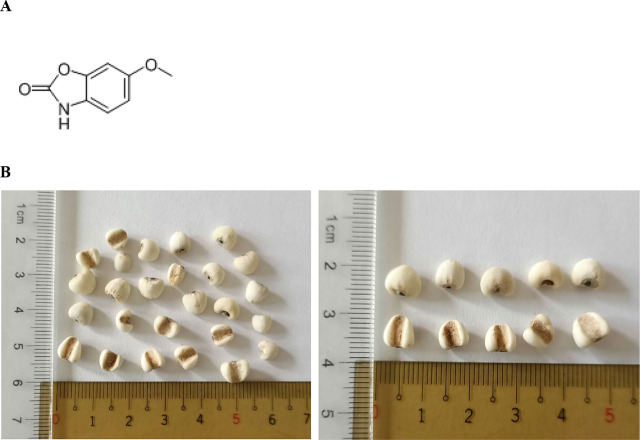
Structure of coixol **(A)** and adlay seeds **(B)**.

Alzheimer’s disease (AD), a common neurodegenerative disorder, is characterized by extracellular accumulation of beta-amyloid (Abeta) peptides [8]. It is documented that Abeta impaired Na^+^-K^+^-ATPase activity, disturbed Ca^2+^ homeostasis, and lessened mitochondrial membrane potential (MMP) in neurons and astrocytes, which in turn led to the loss of mitochondrial biofunctions in neurons and astrocytes [9, 10]. It has been indicated that Abeta activated NF-κB and p38MAPK pathways, and consequently facilitated the generation of oxidative and inflammatory mediators like reactive oxygen species (ROS), interleukin (IL)-1β, IL-6, and tumor necrosis factor (TNF)-α in brain [11, 12]. In addition, Abeta could upregulate the expression of the receptor of advanced glycation end products (RAGEs) [13]. Then, the interaction between Abeta and RAGEs not only aggravated the activation of the above signaling pathways [14], but also promoted the breakage of blood–brain barrier [15]. Apparently, Abeta jeopardizes neuronal cells through multiple actions, which consequently results in malfunctions and disorders in brain and neuronal system. Therefore, any agent with the effects to maintain mitochondrial membrane integrity and decline NF-κB and p38MAPK pathways may potentially alleviate Abeta-induced neurotoxicity and retard AD progression.

Abeta_25-35_ has large β-sheet fibrils and is the most toxic peptide among many Abeta fragments [16]. It has been indicated that Abeta_25-35_ could trigger neuronal cell apoptosis via activating apoptotic factors such as Bax and caspase-3, and evoke oxidative stress-associated neurotoxicity [17]. Thus, Abeta_25-35_ was applied in our present cell line study. The main aim of this study was to assess the preventive effects of coixol at different concentrations against Abeta_25-35_-induced damage in nerve growth factor (NGF)-differentiated PC12 cells. The influence of coixol upon cell survival, mitochondrial functions, and the variation of oxidative and inflammatory indicators was determined. In addition, the impact of coixol on the mRNA expression and protein production of associated factors such as Bcl-2 and RAGE was evaluated. These results could benefit our understanding about the possibility of using coixol as an anti-AD agent.

## Materials and methods

Coixol (97.5%) obtained from Chem-Impex Int. Inc. was dissolved in dimethyl sulfoxide (DMSO) and then diluted with culture medium. The final concentration of DMSO was lower than 0.5%. NGF at 99% was purchased from Wako Chemical Co. Abeta_25-35_ was obtained from Sigma Chem. Co. and diluted with 10 mM sodium phosphate buffer (PBS, pH 7.2) for experiments. Other materials, including medium and antibiotics, used to prepare cell culture were purchased from Difco Lab.

### Cell culture

Dulbecco’s modified Eagle’s medium (DMEM) was adopted for PC12 cells culture. This medium contained heat-inactivated calf serum (10%), fetal bovine serum (5%), penicillin (100 units/mL), and streptomycin (100 units/mL). Cell culture was processed under air–CO_2_ (95:5) at 37 °C. Cells at 10^5^/mL were treated with NGF at 50 ng/mL, followed by incubation at 37 °C for 5 days to allow differentiation. After washed twice with serum-free DMEM, differentiated cells were collected in 96-well plates for further cultivation.

### Experimental design

**Figure 2.**
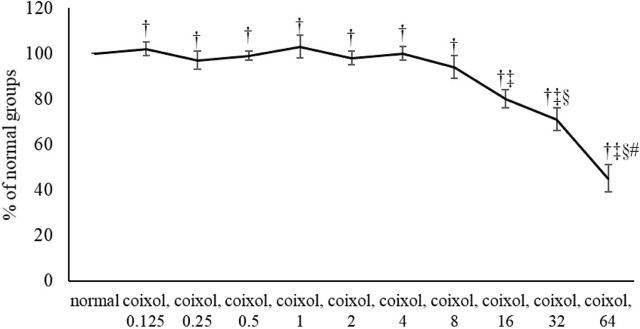
Effects of coixol alone upon cell viability. NGF-differentiated PC12 cells were treated with coixol at 0.125 μM, 0.25 μM, 0.5 μM, 1 μM, 2 μM, 4 μM, 8 μM, 16 μM, or 32 μM for 48 h. Normal group had no coixol. Data are expressed as mean ± SD (n = 8). †: *P* < 0.05 compared the normal; ‡: *P* < 0.05 compared coixol 8; §: *P* < 0.05 compared coixol 16; #: *P* < 0.05 compared coixol 32. NGF, nerve growth factor; SD, standard deviation.

As shown in **Figure 2**, coixol treatments at ≥16 μM resulted in significant cell loss. Thus, the concentrations of coixol used in this study were 0.125–2 μM. Normal groups were cells without coixol or Abeta_25-35_. Cells without coixol, but with Abeta_25-35_, were Abeta groups. Cells were treated with coixol at 0.125 μM, 0.25 μM, 0.5 μM, 1 μM, and 2 μM at 37 °C for 48 h. Subsequently, cells were exposed to Abeta_25-35_ at 20 μM for 24 h.

### Assay of cell survival

Cells at 10^5^/mL was mixed with 3-(4,5-dimethylthiazol-2-yl)-2, 5-diphenyltetrazolium bromide (MTT) at 0.25 mg/mL, followed by incubation at 37 °C for 3 h. MTT formazan product was quantified by a microplate reader (Bio-Rad, Model 550) to read the absorbance at 570 nm. Cell survival was shown as a percentage of normal groups.

### Determination of MMP, Na^+^-K^+^ ATPase, and caspase-3 activities

Cells at 10^5^/mL were allowed to react with Rhodamine 123 at 100 μg/L, followed by incubation at 37 °C for 30 min. Mean fluorescence intensity (MFI) value, acting as an MMP indicator, was assayed by a flow cytometry (Beckman Coulter, FC500 Model). Na^+^-K^+^ ATPase activity was measured by monitoring inorganic phosphate released from ATP. After centrifugation at 5,000 ×*g* and 4 °C for 10 min, cell pellet was collected. Mitochondria isolation kit obtained from Sigma-Aldrich Co. was applied to separate mitochondrial fraction from pellet. Those mitochondria were further mixed with 30 mM Tris–HCl buffer solution (pH 7.4). ATP was added to initiate the assay process, followed by incubation at 37 °C for 15 min. Trichloroacetic acid (15%) was added to stop assay. The absorbance at 640 nm was monitored using a fluorophotometer. Caspase-3 activity was determined by an assay kit (Upstate). In brief, cells at 10^5^/mL were mixed with 1 mL lysis buffer consisted of 5 mM Tris–HCl, 0.1% Triton X-100, and 20 mM EDTA. Then, this mixture was incubated at 25 °C for 30 min. After centrifugation at 10,000 × *g* and 4 °C for 15 min, supernatant was collected to react with a solution containing *N*-acetyl-Asp-Glu-Val-Asp-*p*-nitroanilide, a specific substrate of caspase-3.

### Measurement of intracellular Ca^2+^ level

After cells were exposed to Abeta_25-35_, intracellular Ca^2+^ level was measured at 0 h, 12 h, and 24 h according to a previously reported method ^[18]^. In brief, cell homogenate was added into a solution containing Fura-2AM, 0.1% DMSO, and 1% bovine serum albumin. After incubation at 37 °C for 30 min in dark condition, fluorescence value was recorded by a Shimadzu spectrofluorimeter (Model RF-5000), in which absorbance was 340 nm and 380 nm and emission was 510 nm. Ca^2+^ level (in nM) was calculated using a formula: *K*_d_ × [(*R*–*R*_min_)/(*R*_max_–*R*)] × FD/FS. *K*_d_ value was 224, and *R* value was the ratio of absorbance values read at 340 nm and 380 nm. *R*_min_ and *R*_max_ were measured by treating cells with ethylene glycol tetraacetic acid and Triton X-100, respectively. FD and FS were the fluorescence values of Ca^2+^-free form and Ca^2+^-bound form detected at 380 nm and 340 nm, respectively.

### Quantification of oxidative and inflammatory factors

The content of glutathione (GSH), and the activities of catalase, glutathione peroxidase (GPX), and glutathione reductase (GR) were quantified by assay kits purchased from OxisResearch Co.. Cell homogenate at 100 μL was allowed to react with 2ʹ,7ʹ-dichlorofluorescein diacetate at 2 mg/mL to detect ROS level. After incubation at 37 °C for 30 min, fluorescence absorbance at 525 nm and 488 nm was recorded by a fluorescence microplate reader (Molecular Devices). Data were directly reported as relative fluorescence units (RFUs)/mg protein. Cell DNA was extracted using a kit purchased from Wako Pure Chem. Co.. The concentration of 8-OHdG, a DNA oxidative marker, was determined using ELISA method. The absorbance (OD = 450 nm) was recorded. The standards used for 8-OHdG measurement were in the range of 0–10 ng/mL. Levels of IL-1β, IL-6, TNF-α, and prostaglandin E_2_ (PGE_2_) were measured by assay kits purchased from Cayman Chem. Co.. Protein concentration of used cell supernatants was analyzed by a BCA protein (Wako Pure Chem. Co., Tokyo, Japan) assay kit.

### Analyses of Bcl-2 and Bax mRNA expression

The concentration of total RNA extracted from cells was assayed by reading the absorbance at 260 nm. RNA at 5 μg was used to synthesize cDNA using a commercial synthesis kit purchased from Legene Biosciences. The synthesized cDNA was applied for real-time polymerase chain reaction (RT-PCR). A SYBR Green I Master kit (Sigma-Aldrich Co.) was used to determine the fluorescence value, in which a RT sequence detection system (ABI 7500 PCR Instrument) was equipped. The primers of target molecules were reported as follows: Bcl-2, forward, 5ʹ-GTG GAT GAC TGA GTA CCT GAA C-3ʹ, reverse, 5ʹ-GAG ACA GCC AGG AGA AAT CAA-3ʹ; Bax, forward, 5ʹ-GCT GAT GGC AAC TTC AAC TG-3ʹ, reverse, 5ʹ-ATC AGC TCG GGC ACT TTA G-3ʹ; and GAPDH (a housekeeping gene), forward, 5ʹ-AGA GGC AGG GAT GTT CTG-3ʹ, reverse, 5ʹ-GAC TCA TGA CCA CAG TCC ATG C-3ʹ.

### Analyses for NF-κB binding activity

NF-κB p50/65 binding activity was evaluated by an assay kit purchased from Chemicon Int. Co.. Extracted nuclear protein at 10 mg was allowed to react with 3,3′,5,5′-tetramethylbenzidine, an antibody of NF-κB p50/p65, followed by incubation at 25 °C for 60 min. Then, horseradish peroxidase-conjugated antibody was added, and this mixture was further incubated for another 60 min. The absorbance (OD = 450 nm) was read, and result was expressed as folds of normal groups.

### Protein production of NF-κB p-p65, p-p38, RAGE, nuclear factor E2-related factor 2 (Nrf2), and inducible nitric oxide synthase (iNOS)

Protein concentration of cell homogenate was determined by reagents purchased from Bio-Rad Laboratories Inc.. Sample with 20 μg protein was used to assess the production of phosphorylated NF-κB p65 (ab207481), phosphorylated p38 (ab207483), RAGE (ab197745), Nrf2 (ab277397), and iNOS (ab253219) by corresponding ELISA kit (Abcam Co.) according to the manufacturer’s instructions.

### Statistical analysis

Data were expressed as mean ± standard deviation (SD). Each value was obtained from eight different preparations (n = 8). Statistical analyses were processed by one-way analysis of variance, and Dunnett’s *t* test was used for post hoc comparison. When *P*-value was lower than 0.05, difference among means was considered significant.

## Results

### Coixol increased cell survival and improved mitochondrial functions

**Figure 3.**
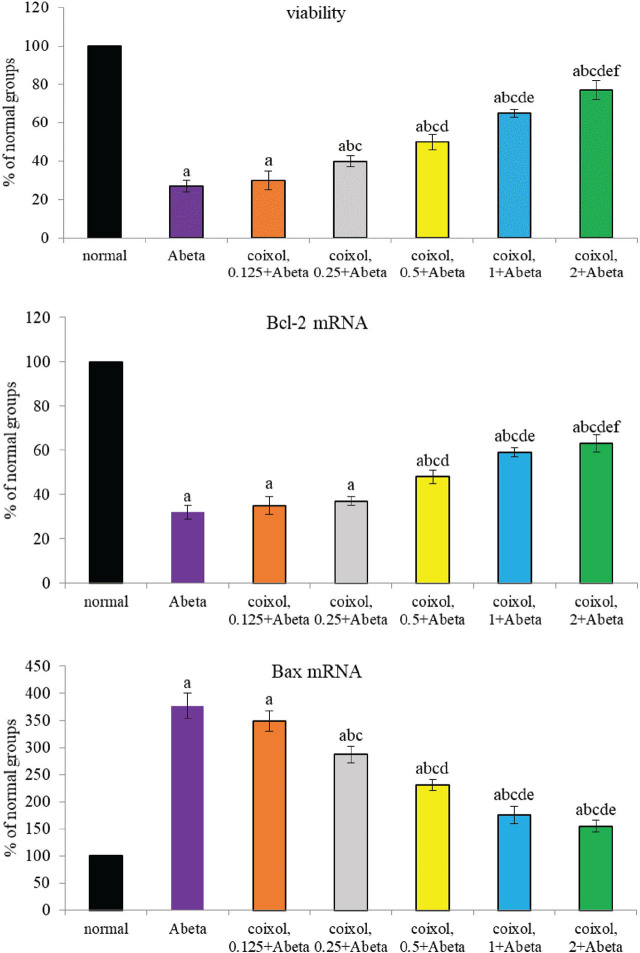
Effects of coixol on cell viability, Bcl-2 mRNA expression, and Bax mRNA expression. NGF-differentiated PC12 cells were pretreated with coixol at 0.125 μM, 0.25 μM, 0.5 μM, 1 μM, or 2 μM for 48 h, followed by Abeta_25-35_ exposure for 24 h. Normal groups had no coixol or Abeta_25-35_. Abeta groups had no coixol, but with Abeta_25-35_. Data are expressed as mean ± SD (n = 8). a: *P* < 0.05 compared the normal; b: *P* < 0.05 compared Abeta; c: *P* < 0.05 compared coixol 0.125 + Abeta; d: *P* < 0.05 compared coixol 0.25 + Abeta; e: *P* < 0.05 compared coixol 0.5 + Abeta; and f: *P* < 0.05 compared coixol 1 + Abeta. NGF, nerve growth factor; SD, standard deviation.

**Figure 4.**
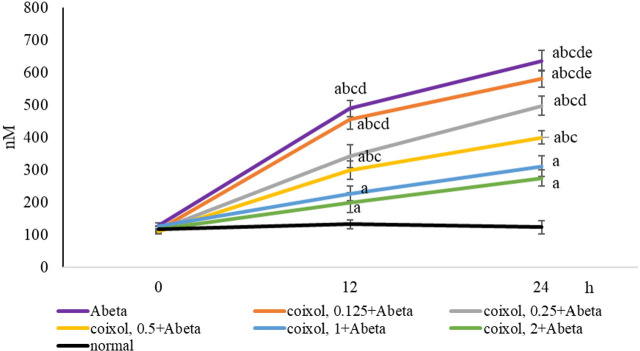
Effects of coixol on intracellular Ca^2+^ level. NGF-differentiated PC12 cells were pretreated with coixol at 0.125 μM, 0.25 μM, 0.5 μM, 1 μM, or 2 μM for 48 h, followed by Abeta_25-35_ exposure for 24 h. Intracellular Ca^2+^ level was measured at 0 h, 12 h, and 24 h. Normal groups had no coixol or Abeta_25-35_. Abeta groups had no coixol, but with Abeta_25-35_. Data are expressed as mean ± SD (n = 8). a: *P* < 0.05 compared the normal; b: *P* < 0.05 compared Abeta; c: *P* < 0.05 compared coixol 0.125 + Abeta; d: *P* < 0.05 compared coixol 0.25 + Abeta; e: *P* < 0.05 compared coixol 0.5 + Abeta; and f: *P* < 0.05 compared coixol 1 + Abeta. NGF, nerve growth factor; SD, standard deviation.

Compared with normal groups, Abeta_25-35_ led to cell death (**Figure 3**, *P* < 0.05). Coixol pretreatments at 0.25–2 μM increased cell survival when compared with Abeta groups (*P* < 0.05). Abeat_25-35_ lowered Bcl-2 mRNA expression and increased Bax mRNA expression. Compared with Abeta groups, coixol pretreatments upregulated Bcl-2 mRNA expression at 0.5–2 μM and downregulated Bax mRNA expression at 0.25–2 μM (*P* < 0.05). Abeta_25-35_ reduced MMP and Na^+^-K^+^ ATPase activity and increased caspase-3 activity (**Table 1**, *P* < 0.05). Compared with Abeta groups, coixol at 0.25–2 μM increased MMP and lowered caspase-3 activity (*P* < 0.05), and coixol at 0.125–2 μM increased Na^+^-K^+^ ATPase activity (*P* < 0.05). Abeta_25-35_ exposure increased intracellular Ca^2+^ level (**Figure 4**, *P* < 0.05). Coixol pretreatments at 0.25–2 μM reduced intracellular Ca^2+^ level at 12 h and 24 h when compared with Abeta groups (*P* < 0.05).

**Table 1.** Effects (% of normal groups) of coixol on MMP, Na^+^-K^+^ ATPase, and caspase-3 activities

	**MMP**	**Na^+^-K^+^ ATPase**	**Caspase-3**
Normal	100	100	100
Abeta	25 ± 5^*^	30 ± 3^*^	337 ± 19^*^
Coixol, 0.125 + Abeta	29 ± 3^*^	39 ± 2^*^^,^^**^	315 ± 23^*^
Coixol, 0.25 + Abeta	38 ± 4^*^^,^^**^^,^^***^	44 ± 5^*^^,^^**^	276 ± 11^*^^,^^**^^,^^***^
Coixol, 0.5 + Abeta	49 ± 3^*^^,^^**^^,^^***^^,^^#^	56 ± 2^*^^,^^**^^,^^***^^,^^#^	230 ± 13^*^^,^^**^^,^^***^^,^^#^
Coixol, 1 + Abeta	60 ± 5^*^^,^^**^^,^^***^^,^^#^^,^^##^	68 ± 3^*^^,^^**^^,^^***^^,^^#^^,^^##^	183 ± 8^*^^,^^**^^,^^***^^,^^#^^,^^##^
Coixol, 2 + Abeta	71 ± 6^*^^,^^**^^,^^***^^,^^#^^,^^##^^,^^###^	74 ± 5^*^^,^^**^^,^^***^^,^^#^^,^^##^	172 ± 12^*^^,^^**^^,^^***^^,^^#^^,^^##^

NGF-differentiated PC12 cells were pretreated with coixol at 0.125 μM, 0.25 μM, 0.5 μM, 1 μM, or 2 μM for 48 h, followed by Abeta_25-35_ exposure for 24 h. Normal groups had no coixol or Abeta_25-35_. Abeta groups had no coixol, but with Abeta_25-35_. Data are expressed as mean ± SD (n = 8).

* *P* < 0.05 compared the normal.

** *P* < 0.05 compared Abeta.

*** *P* < 0.05 compared coixol 0.125 + Abeta.

# *P* < 0.05 compared coixol 0.25 + Abeta.

## *P* < 0.05 compared coixol 0.5 + Abeta.

### *P* < 0.05 compared coixol 1 + Abeta.

MMP, mitochondrial membrane potential; NGF, nerve growth factor SD, standard deviation.

### Coixol diminished oxidative and inflammatory stress

**Figure 5.**
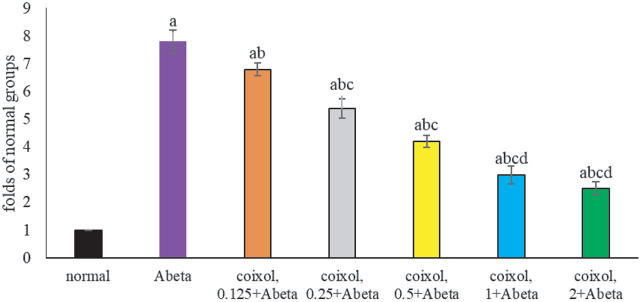
Effects of coixol on NF-κB binding activity. NGF-differentiated PC12 cells were pretreated with coixol at 0.125 μM, 0.25 μM, 0.5 μM, 1 μM, or 2 μM for 48 h, followed by Abeta_25-35_ exposure for 24 h. Normal groups had no coixol or Abeta_25-35_. Abeta groups had no coixol, but with Abeta_25-35_. Data are expressed as mean ± SD (n = 8). a: *P* < 0.05 compared the normal; b: *P* < 0.05 compared Abeta; c: *P* < 0.05 compared coixol 0.125 + Abeta; d: *P* < 0.05 compared coixol 0.25 + Abeta; e: *P* < 0.05 compared coixol 0.5 + Abeta; and f: *P* < 0.05 compared coixol 1 + Abeta. NF-κB, nuclear factor kappa B; NGF, nerve growth factor; SD, standard deviation.

Abeta_25-35_ increased ROS and 8-OHdG levels, lowered GSH content, and decreased GPX, GR, and catalase activities (**Table 2**, *P* < 0.05). Compared with Abeta groups, coixol pretreatments at 0.25–2 μM lowered ROS and 8-OHdG levels, increased GSH content, and increased GPX and GR activities (*P* < 0.05). Coixol pretreatments at 0.125–2 μM increased catalase activity (*P* < 0.05). As shown in **Table 3**, Abeta_25-35_ increased the release of TNF-α, IL-1β, IL-6, and PGE_2_ (*P* < 0.05). Compared with Abeta groups, coixol pretreatments at 0.125–2 μM decreased the levels of these four cytokines (*P* < 0.05). Abeta_25-35_ increased the NF-κB binding activity by 6.8-fold (**Figure 5**, *P* < 0.05). Compared with Abeta groups, coixol pretreatments at 0.125–2 μM lowered NF-κB binding activity (*P* < 0.05). However, coixol pretreatments at 2 μM did not cause further decline in the level of four inflammatory cytokines and NF-κB binding activity when compared with coixol pretreatments at 1 μM (*P* > 0.05).

**Table 2.** Effects of coixol on the levels or activities of ROS, 8-OHdG, GSH, GPX, GR, and catalase

	**ROS RFU/mg protein**	**8-OHdG ng/mg protein**	**GSH ng/mg protein**	**GPX U/mg protein**	**GR U/mg protein**	**Catalase U/mg protein**
Normal	0.13 ± 0.04	0.43 ± 0.06	88 ± 5	72.1 ± 1.7	62.8 ± 1.1	70.4 ± 1.9
Abeta	3.11 ± 0.21^*^	2.72 ± 0.10^*^	26 ± 2^*^	30.2 ± 0.9^*^	27.6 ± 0.7^*^	24.1 ± 1.0^*^
Coixol, 0.125 + Abeta	2.72 ± 0.14^*^	2.51 ± 0.15^*^	29 ± 4^*^	32.5 ± 1.2^*^	29.0 ± 1.2^*^	30.5 ± 0.8^*^^,^^**^
Coixol, 0.25 + Abeta	2.15 ± 0.08^*^^,^^**^^,^^***^	2.02 ± 0.07^*^^,^^**^^,^^***^	36 ± 3^*^^,^^**^^,^^***^	43.1 ± 1.0^*^^,^^**^^,^^***^	37.1 ± 1.0^*^^,^^**^^,^^***^	39.3 ± 1.2^*^^,^^**^^,^^***^
Coixol, 0.5 + Abeta	1.64 ± 0.15^*^^,^^**^^,^^***^^,^^#^	1.73 ± 0.11^*^^,^^**^^,^^***^^,^^#^	49 ± 5^*^^,^^**^^,^^***^^,^^#^	52.4 ± 1.3^*^^,^^**^^,^^***^^,^^#^	45.3 ± 0.9^*^^,^^**^^,^^***^^,^^#^	48.4 ± 1.5^*^^,^^**^^,^^***^^,^^#^
Coixol, 1 + Abeta	1.03 ± 0.17^*^^,^^**^^,^^***^^,^^#^^,^^##^	1.36 ± 0.08^*^^,^^**^^,^^***^^,^^#^^,^^##^	60 ± 4^*^^,^^**^^,^^***^^,^^#^^,^^##^	55.0 ± 2.1^*^^,^^**^^,^^***^^,^^#^	53.2 ± 1.4^*^^,^^**^^,^^***^^,^^#^^,^^##^	55.9 ± 1.1^*^^,^^**^^,^^***^^,^^#^^,^^##^
Coixol, 2 + Abeta	0.87 ± 0.09^*^^,^^**^^,^^***^^,^^#^^,^^##^	1.13 ± 0.13^*^^,^^**^^,^^***^^,^^#^^,^^##^	71 ± 3^*^^,^^**^^,^^***^^,^^#^^,^^##^^,^^###^	62.7 ± 1.4^*^^,^^**^^,^^***^^,^^#^	55.3 ± 0.8^*^^,^^**^^,^^***^^,^^#^^,^^##^	60.3 ± 0.9^*^^,^^**^^,^^***^^,^^#^^,^^##^

NGF-differentiated PC12 cells were pretreated with coixol at 0.125 μM, 0.25 μM, 0.5 μM, 1 μM, or 2 μM for 48 h, followed by Abeta_25-35_ exposure for 24 h. Normal groups had no coixol or Abeta_25-35_. Abeta groups had no coixol, but with Abeta_25-35_. Data are expressed as mean ± SD (n = 8).

* *P* < 0.05 compared the normal.

** *P* < 0.05 compared Abeta.

*** *P* < 0.05 compared coixol 0.125 + Abeta.

# *P* < 0.05 compared coixol 0.25 + Abeta.

## *P* < 0.05 compared coixol 0.5 + Abeta.

### *P* < 0.05 compared coixol 1 + Abeta.

GPX, glutathione peroxidase; GR, glutathione reductase; GSH, glutathione; NGF, nerve growth factor; RFU, relative fluorescence unit; ROS, reactive oxygen species; SD, standard deviation.

### Coixol regulated protein production of associated factors

**Figure 6.**
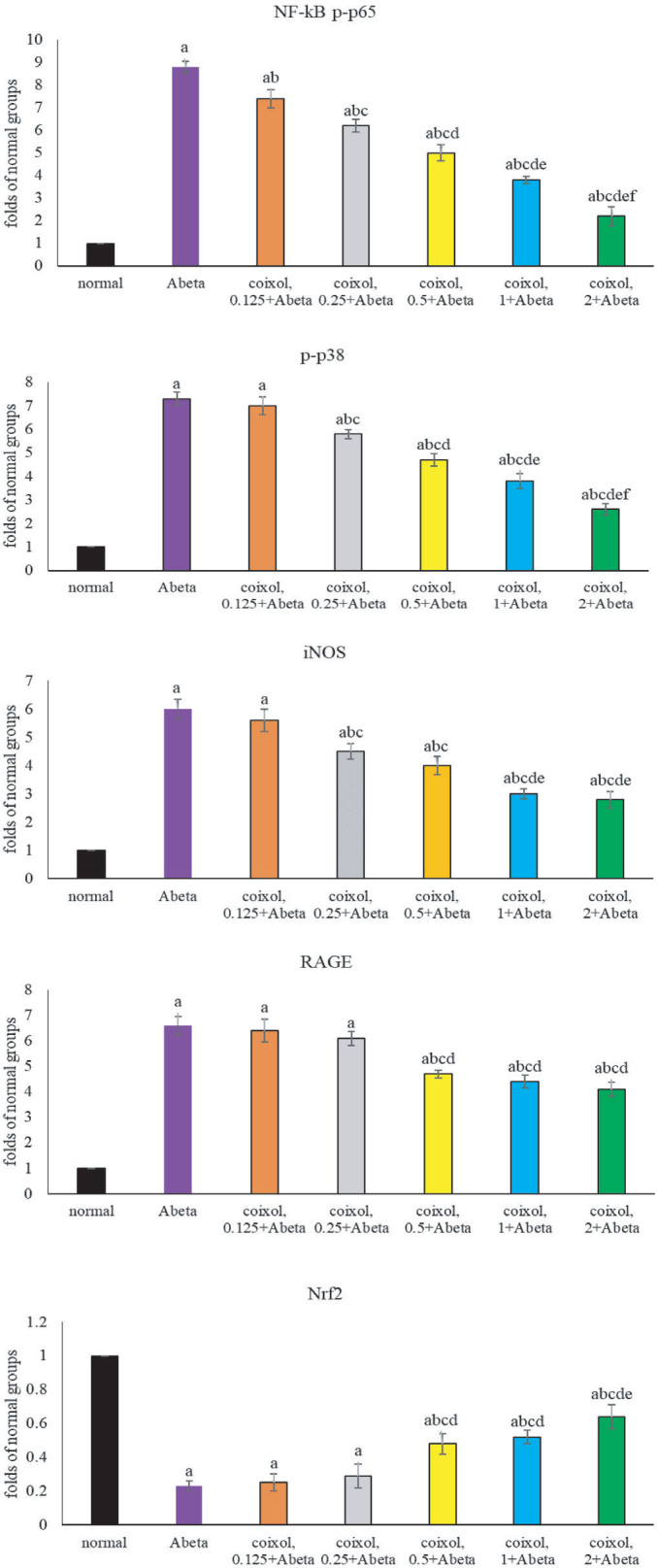
Effects of coixol on protein expression of phosphorylated NF-κB p65, phosphorylated p38, iNOS, RAGE, and Nrf2. NGF-differentiated PC12 cells were pretreated with coixol at 0.125 μM, 0.25 μM, 0.5 μM, 1 μM, or 2 μM for 48 h, followed by Abeta_25-35_ exposure for 24 h. Normal groups had no coixol or Abeta_25-35_. Abeta groups had no coixol, but with Abeta_25-35_. Data are expressed as mean ± SD (n = 8). a: *P* < 0.05 compared the normal; b: *P* < 0.05 compared Abeta; c: *P* < 0.05 compared coixol 0.125 + Abeta; d: *P* < 0.05 compared coixol 0.25 + Abeta; e: *P* < 0.05 compared coixol 0.5 + Abeta; and f: *P* < 0.05 compared coixol 1 + Abeta. iNOS, inducible nitric oxide synthase; NF-κB, nuclear factor kappa B; NGF, nerve growth factor; RAGE, receptor of advanced glycation end product; SD, standard deviation.

As shown in **Figure 6**, Abeta_25-35_ increased phosphorylated products of NF-κB p65 and p38; upregulated iNOS and RAGE protein production; and downregulated Nrf2 protein generation (*P* < 0.05). Compared with Abeta groups, coixol pretreatments at 0.125–2 μM suppressed NF-κB p65 phosphorylation (*P* < 0.05), and at 0.25–2 μM they decreased p38 phosphorylation and iNOS protein production (*P* < 0.05). Coixol pretreatments at 0.5–2 μM lowered RAGE protein generation and increased Nrf2 protein formation (*P* < 0.05).

## Discussion

Abeta_25-35_ is a toxic peptide. It has been documented that Abeta_25-35_ causes neurotoxicity and facilitates AD deterioration through multiple ways, including disturbance of neuronal cell biomembranes, loss of mitochondrial integrity, activation of signaling pathways, and overproduction of oxidants and inflammatory cytokines ^[19, 20]^. In our present study, this toxic peptide was adopted to induce damage to NGF-differentiated PC12 cells. We found that coixol pretreatments effectively protected NGF-differentiated PC12 cells against Abeta_25-35_-induced apoptotic, oxidative, and inflammatory damage, as well as increased cell survival. It has been reported that coixol has antimicrobial, antioxidative, antidiabetic and anti-inflammatory activities [4, 7, 21]. The data from our present study, an AD cell model, extended the bio-activities of coixol to anti-AD. Mitochondrial dysfunction played a crucial role in pathogenic cellular progression of AD because mitochondrial dysfunction launched apoptotic responses, promoted oxidative stress and gave rise to intracellular calcium deregulation [22]. Thus, any improvement upon mitochondrial functions has been considered as an effective strategy for prevention or treatment of AD [23]. The study of Graham et al. [24] and our present study revealed that Abeta_25-35_ evoked mitochondrial mediated apoptotic pathway, which were evidenced by greater Bax mRNA expression and less Bcl-2 mRNA expression. In addition, we found that Abeta_25-35_ caused mitochondrial disorders including lower MMP, impaired Na^+^-K^+^ ion exchange, disturbed Ca^2+^ homeostasis and higher caspase-3 activity. Furthermore, our data revealed that coixol treatments markedly mediated these apoptotic and antiapoptotic factors, stabilized mitochondrial membranes, maintained ions homeostasis and limited caspase-3 activity, which consequently benefited mitochondrial biofunctions and favored NGF-differentiated PC12 cells survival. These findings indicated that coixol could protect mitochondria to counteract or block Abeta_25-35_-induced mitochondrial damage. Since coixol had benefited mitochondrial biofunctions, the observed attenuation in oxidative injury in coixol-treated cells could be partially elucidated.

Dysregulation of neuronal Ca^2+^ homeostasis is a key responsible for the pathogenesis of AD because disrupted Ca^2+^ not only induces synaptic deficits but also augments the deposit of Abeta plaques [25]. Meanwhile, the increased mitochondrial Ca^2+^ disruption promoted neuronal excitotoxicity and even neuronal cell death [26]. In our present study, Abeta_25-35_ enhanced intracellular Ca^2+^ release, which indicated that Abeta_25-35_ had impaired mitochondrial Ca^2+^ homeostasis. However, we found that coixol treatment at 0.25–2 μM effectively reduced intracellular Ca^2+^ level. These findings suggest that coixol could penetrate cells and keep the integrity of biomembranes, which in turn benefited Ca^2+^ homeostasis.

Abeta_25-35_ accelerated oxidative and inflammatory responses in brain of AD-like rodents and PC12 cells [27, 28]. Our data were contradictory to those of previous studies because Abeta_25-35_ exposure resulted in GSH depletion and less activities of GPX, GR, and catalase, as well as massive production of ROS and 8-OHdG. Coixol treatments markedly decreased oxidant production and retained antioxidative defensive capability, which subsequently weakened Abeta_25-35_-induced oxidative stress in NGF-differentiated PC12 cells. On the contrary, Abeta_25-35_ exposure obviously elevated inflammatory stress via stimulating the release of inflammatory mediators such as TNF-α, PGE_2_, IL-1β, and IL-6. However, coixol treatments substantially decreased the generation of these cytokines and ameliorated inflammatory stress in those cells. Consequently, the improvement in oxidative and inflammatory stress due to coixol contributed to greater survival of NGF-differentiated PC12 cells. Our data showed that coixol treatments increased the antioxidative and anti-inflammatory protection of neuronal cells against Abeta_25-35_.

Abeta_25-35_ enhanced NF-κB binding activity and protein production of NF-κB p-p65, p-p38, iNOS, and RAGE, which led to the massive generation of downstream oxidative and inflammatory factors, and consequently facilitated the progression of AD [29, 30]. Our data revealed that coixol treatments markedly lessened NF-κB binding activity and limited protein production of NF-κB p-p65, p-p38, iNOS, and RAGE. These findings implied that coixol was able to suppress NF-κB, p38MAPK, iNOS, and RAGE pathways, and contributed to mitigate both oxidative and inflammatory responses. Nrf2, a key transcription element, could affect the gene expression of endogenous antioxidants such as GPX and catalase [31]. Abeta_25-35_ exposure downregulated Nrf2 protein expression and restricted the formation of its downstream enzymatic antioxidants [32]. Thus, the observed lower activity of GPX, GR, and catalase in Abeta_25-35_-treated cells as we observed could be partially elucidated. However, we found that coixol increased Nrf2 protein production, which benefited antioxidative defense capability of those cells. It is highly possible that coixol mediated several upstream oxidative- and inflammatory-associated elements or pathways, which subsequently decreased the formation of oxidants and inflammatory cytokines, and finally improved the viability of NGF-differentiated PC12 cells. It is interesting to note that coixol at 0.125 μM reduced the levels of TNF-α, IL-1β, IL-6, and PGE_2_ (**Table 3**), but at this concentration it failed to affect protein level of p-p38 and iNOS (**Figure 5**). Thus, besides mediating these examined signaling pathways, coixol might be able to execute other antioxidative and anti-inflammatory actions. On the contrary, we found that coixol could restrict RAGE generation. This agent might be able to improve other glycation-associated diseases, such as diabetes, because RAGE plays a crucial pathological role in those diseases.

**Table 3.** Effects (pg/mg protein) of coixol on the levels of TNF-α, IL-1β, IL-6, and PGE_2_

	**TNF-α**	**IL-1**β	**IL-6**	**PGE**_2_
Normal	12 ± 3	7 ± 2	9 ± 3	109 ± 8
Abeta	261 ± 14^*^	199 ± 13^*^	204 ± 9^*^	442 ± 23^*^
Coixol, 0.125 + Abeta	229 ± 10^*^^,^^**^	162 ± 11^*^^,^^**^	170 ± 10^*^^,^^**^	386 ± 17^*^^,^^**^
Coixol, 0.25 + Abeta	185 ± 9^*^^,^^**^^,^^***^	120 ± 8^*^^,^^**^^,^^***^	131 ± 8^*^^,^^**^^,^^***^	319 ± 12^*^^,^^**^^,^^***^
Coixol, 0.5 + Abeta	136 ± 12^*^^,^^**^^,^^***^^,^^#^	84 ± 10^*^^,^^**^^,^^***^^,^^#^	97 ± 6^*^^,^^**^^,^^***^^,^^#^	257 ± 20^*^^,^^**^^,^^***^^,^^#^
Coixol, 1 + Abeta	99 ± 7^*^^,^^**^^,^^***^^,^^#^^,^^##^	61 ± 6^*^^,^^**^^,^^***^^,^^#^^,^^##^	72 ± 5^*^^,^^**^^,^^***^^,^^#^^,^^##^	192 ± 8^*^^,^^**^^,^^***^^,^^#^^,^^##^
Coixol, 2 + Abeta	90 ± 5^*^^,^^**^^,^^***^^,^^#^^,^^##^	52 ± 4^*^^,^^**^^,^^***^^,^^#^^,^^##^	68 ± 4^*^^,^^**^^,^^***^^,^^#^^,^^##^	177 ± 9^*^^,^^**^^,^^***^^,^^#^^,^^##^

NGF-differentiated PC12 cells were pretreated with coixol at 0.125 μM, 0.25 μM, 0.5 μM, 1 μM, or 2 μM for 48 h, followed by Abeta_25-35_ exposure for 24 h. Normal groups had no coixol or Abeta_25-35_. Abeta groups had no coixol, but with Abeta_25-35_. Data are expressed as mean ± SD (n = 8).

* *P* < 0.05 compared the normal.

** *P* < 0.05 compared Abeta.

*** *P* < 0.05 compared coixol 0.125 + Abeta.

# *P* < 0.05 compared coixol 0.25 + Abeta.

## *P* < 0.05 compared coixol 0.5 + Abeta.

### *P* < 0.05 compared coixol 1 + Abeta.

IL-1β, interleukin-1 beta; IL-6, interleukin-6; NGF, nerve growth factor; PGE_2_, prostaglandin E_2_; SD, standard deviation; TNF-α, tumor necrosis factor-α.

Coixol is a small polyphenolic compound with a molecular weight of 165.15. It is highly possible that this compound could penetrate neuronal cells and execute its bioactivities for those cells. Based on its natural property, the application of this agent for AD’s prevention and/or improvement might be safe. However, it is reported that coixol exhibited cytotoxic effects on Chang liver cells than on normal liver cells [33]. We also found that coixol treatments at higher than 16 mM resulted in loss of NGF-differentiated PC12 cells (**Figure 2**). Thus, further animal studies are necessary to prove the effects, action modes, appropriate doses, and safety of this agent for AD’s prevention or treatment. In addition, coixol is an active compound of adlay seeds, and it has been documented that adlay seeds has many nutritional and medicinal advantages [34, 35]. Our data support that coixol contributes to the observed healthy profits of adlay seeds.

## Conclusions

Coixol displayed multiple preventive activities in NGF-differentiated PC12 cells against Abeta_25-35_. This agent could mitigate Abeta_25-35_-derived apoptotic, oxidative, and inflammatory stress, as well as mediate related pathways in this AD cell model. These findings implied that coixol was a potent anti-AD agent. Since coixol is an active compound of adlay seeds, the consumption of adlay seeds might also benefit prevention and/or alleviation of AD.
